# Calorimetry, physicochemical characteristics and nitrogen release from extruded urea

**DOI:** 10.1038/s41598-021-97886-0

**Published:** 2021-09-15

**Authors:** Noemila Debora Kozerski, Luís Carlos Vinhas Ítavo, Camila Celeste Brandão Ferreira Ítavo, Gelson dos Santos Difante, Alexandre Menezes Dias, Lincoln Carlos Silva de Oliveira, Elias Nogueira de Aguiar, Alexandre Guimarães Inácio, Antonio Leandro Chaves Gurgel, Geraldo Tadeu dos Santos

**Affiliations:** Faculty of Veterinary Medicine and Animal Science, Federal University of Mato Grosso Do Sul (UFMS), Av. Senador Filinto Muller, 2443. Vila Ipirang, Campo Grande, MS 79070-900 Brazil

**Keywords:** Chemistry, Materials science, Physics

## Abstract

Our hypothesis was that extrusion of urea associated with corn may reduce N solubilization and increase the nutritional quality of this food for ruminants. We aimed to physically and chemically characterize a corn and urea mixture before and after the extrusion process. It was evaluated morphological differences by scanning electron microscopy, nitrogen solubilization, and compound mass loss by thermogravimetry. In scanning electron microscopy, extruded urea showed agglomerated and defined structures, with changes in the morphology of starch granules and urea crystals, differing from the arrangement of the corn and urea mixture. The extruded urea maintained a constant nitrogen release pattern for up to 360 min. In thermogravimetry, extruded urea presented a higher temperature to initiate mass loss, that is, the disappearance of the material with increasing temperature, but the mass loss was lower when compared to the first event of the corn and urea mixture. In conclusion the process of extrusion of urea with corn modifies the original structures of these ingredients and controls the release of nitrogen from the urea, maintaining in its formation an energy source optimizing the use of nitrogen by ruminal bacteria, because the more synchronized the release of starch (energy) and nitrogen, the better the use by ruminal microorganisms.

## Introduction

The use of urea as a source of non-protein nitrogen (NNP) for ruminants is widespread and has been universally accepted as a cheap ingredient to replace plant protein sources in ruminant diets^1^, as ruminal bacteria have the ability to convert ammonia (NH_3_) to high biological value microbial protein when in sync with available energy^2^.

With the increased use of concentrates and urea in the ruminant diet, studies intensified concerning the development of products that controlled the release of N from urea and an extruded starch product was developed, cereals with urea^3,4^ and a 45% protein equivalent^5^. The extrusion process consists of the fusion of urea with the gelatinized starch molecule, upon exposure to pressure, temperature, and humidity, for a certain time, thus obtaining the extruded urea called starea. Urea changes from a crystalline to a non-crystalline structure, which is found within the gelatinized portion of starch^3–5^.

This practice can increase the speed of rumen starch fermentation and reduce the intensity of ammonia release from urea, synchronizing both factors for microbial protein synthesis^6^ and increase the acceptability of urea by animals in concentrates^7^. Improvement of extruded urea production techniques, increase of protein equivalent to 200% crude protein (CP) levels, and utilization for various productive purposes in ruminants has intensified, demanding research aiming to characterize the modifications that processing promotes in the ingredients used, as the demand for use of alternative products is increasing in ruminant diets.

However, comparisons between urea and starea (extruded urea) on nitrogen release in lambs^8,9^ or in lactating cows^10^ have presented different results, not allowing a conclusion on the subject. It is known that in finishing steers a lower concentration of ruminal ammonia is observed in the treatment with starea when compared to a mixture of urea plus coarse sorghum after seven days of feeding^11^. Thus, the hypothesis of this study is that extruded urea may reduce N solubilization compared to urea mixed with maize. Therefore, the objective was to evaluate the characteristics of extruded urea and compare them with the corn and urea mixture before the extrusion process.

## Results

### Scanning electron microscopy (SEM)

**Figure 1:**
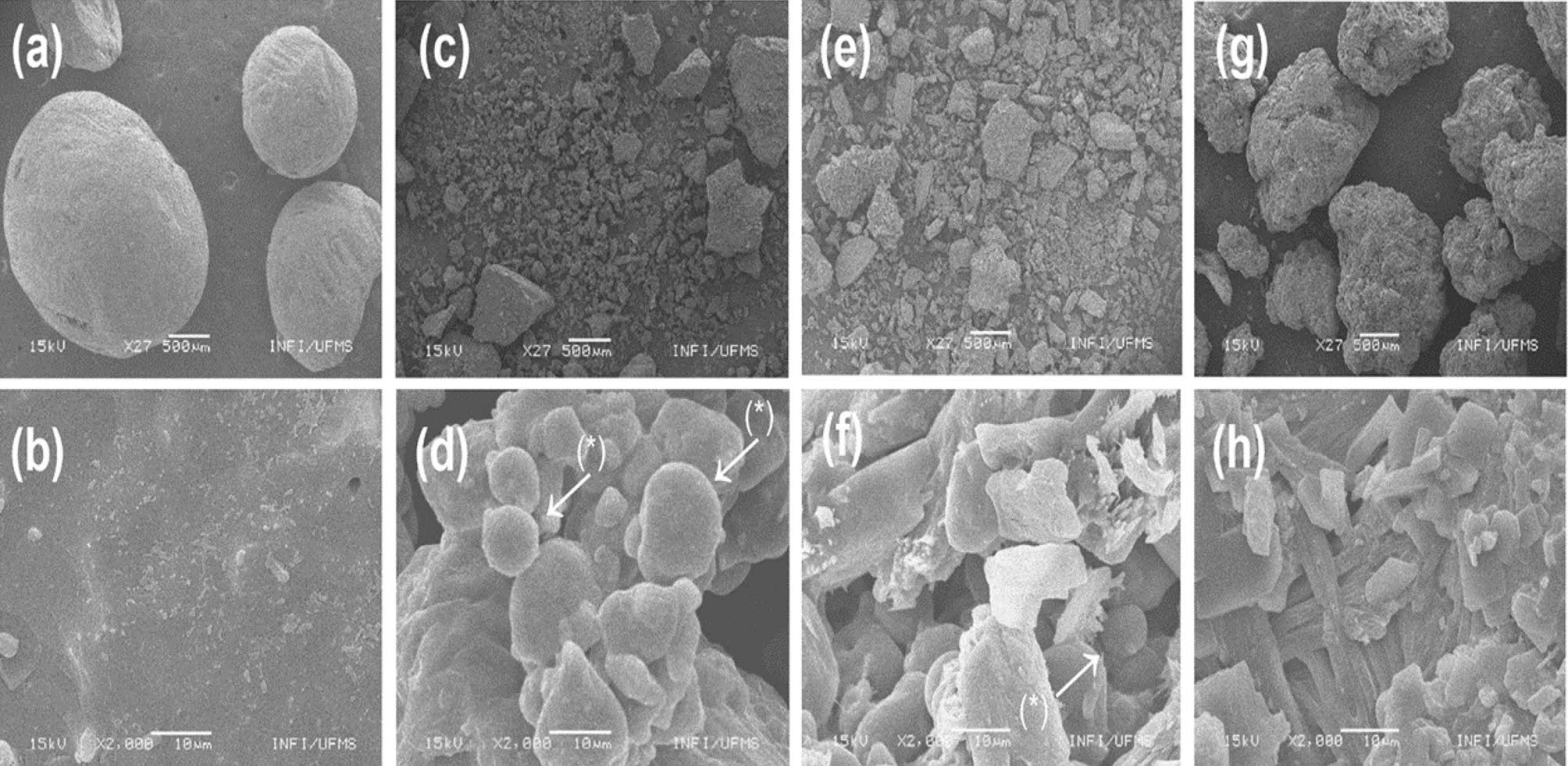
Scanning electron microscopy at 27 and 2000 times increases in urea (**a** and **b**), ground corn (**c** and **d**), corn and urea mixture (**e** and **f**), and extruded urea (**g** and **h**). (*) Starch granules.

Urea has in its original form spherical shapes (Fig. 1a and b). The ground whole corn, is configured in a dispersed structure, being possible to evidence the starch granules, defined by rounded shapes (Fig. 1c and d). The mixture of corn and urea is dispersed in the carbon strip, without having a definite shape, with the presence of starch granules, defined by rounded shapes, and gleaming crystalline structures of urea (Fig. 1e and f). In the extruded urea sample, structures in defined shapes and agglomerates are observed, which formed a complex structure (Fig. 1g and h).

### In vitro nitrogen solubility

**Figure 2:**
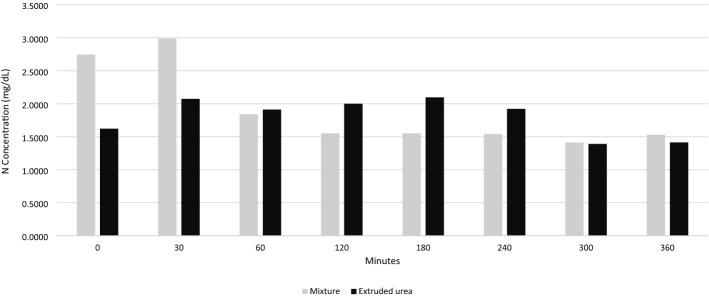
Nitrogen solubility (mg/dL) in water as a function of in vitro incubation time at 39 °C.

The N release peak of the maize and urea sample (Fig. 2) occurred within 30 min (Y_corn_and_urea_mixture_ = 2.8219–0; 2059x; R^2^ = 0.6722).

The reduction in the rate of nitrogen solubilization in the aqueous medium of the extruded urea sample (Fig. 2) evidenced the slower solubilization of N compared to the corn and urea mixture sample (Y_extruded_urea_ = 2.0578–0.0565x; R^2^ = 0.2313).

### Thermogravimetry and derived thermogravimetry

The urea sample was characterized in the TG and DTG curves with five simultaneous stages, that is, the reactions of an event do not end with beginning of the next event. This is evident in the first and second stages, which initiate the decomposition of urea between temperatures of approximately 133.0 and 180.5 C, with loss of most mass, estimated at 72.97% (Table 2). These stages were characterized by the fast and narrow peak DTG (Fig. 4).

Stages three and four (Table 2), characterized by DTG peaks, maintained the concurrency with milder mass loss than in the first and second stages. The fifth stage presented a wider peak in the DTG (Fig. 4), which characterizes the end of urea decomposition.

**Figure 3:**
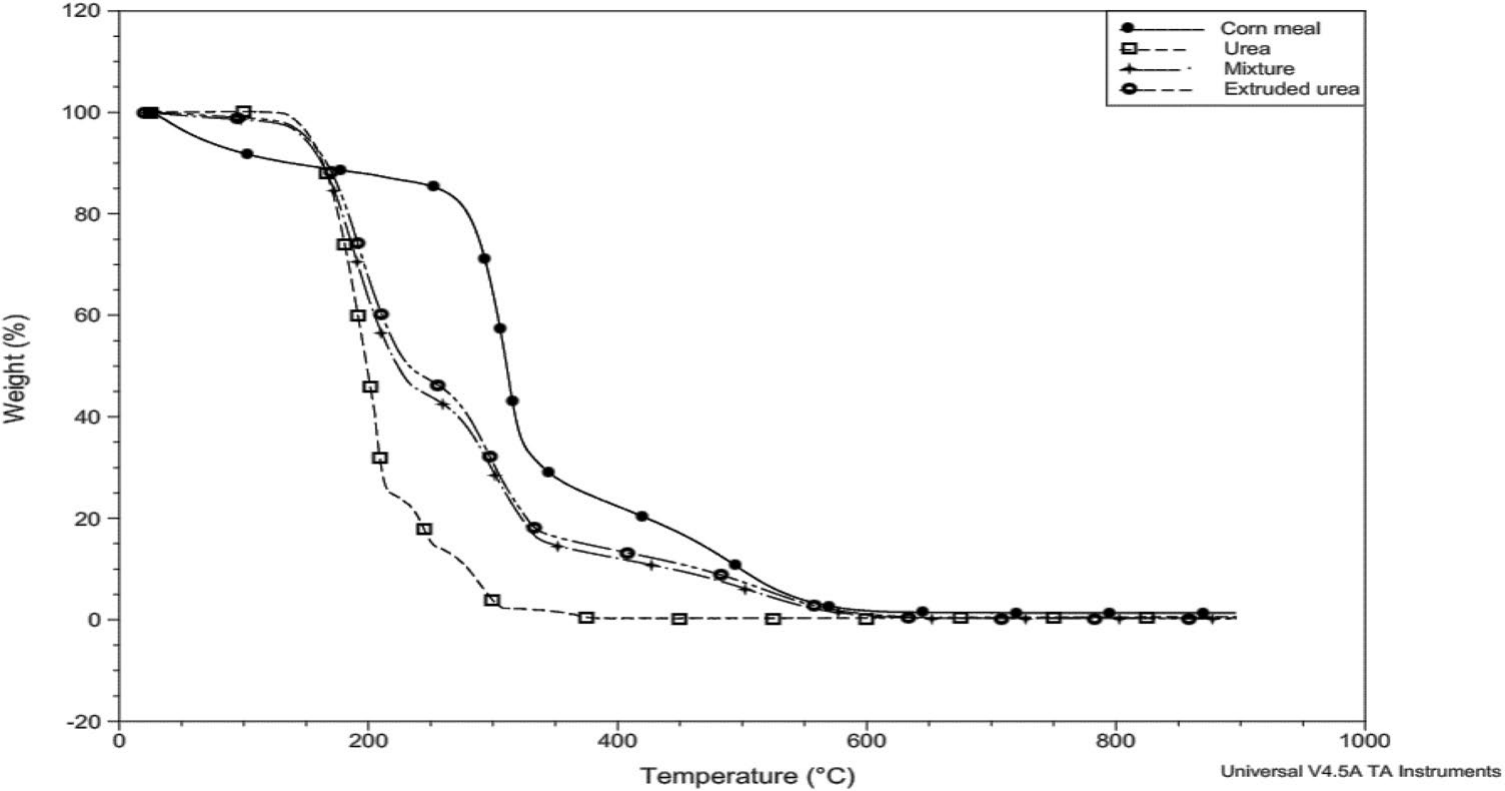
Overlapping TG curves of urea, ground corn, mix corn and urea, and extruded urea.

**Figure 4:**
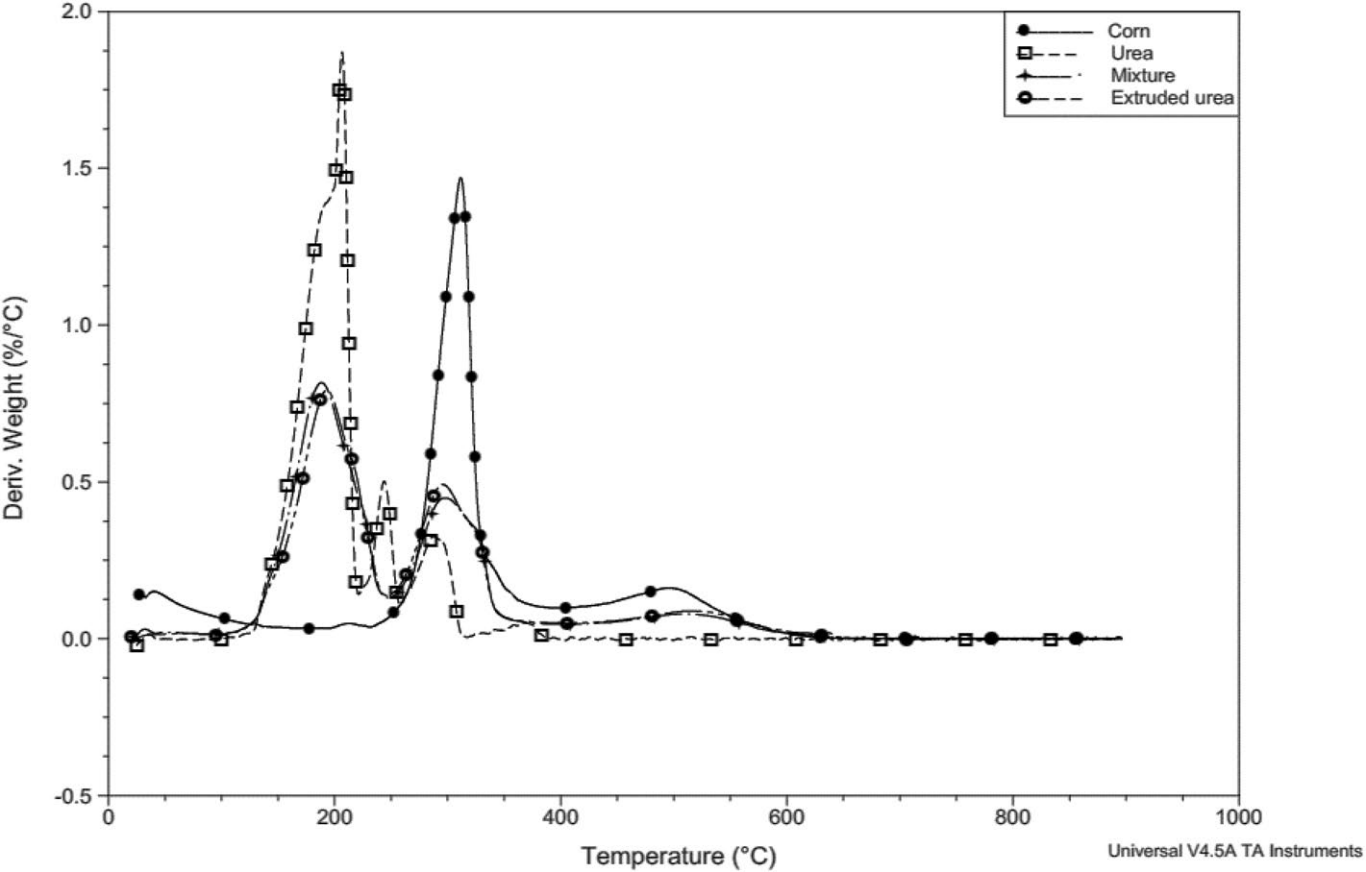
Overlapping DTG curves of urea, ground corn, mix corn and urea, and extruded urea.

The sample of ground whole corn showed no stability (Table 2). The second stage was characterized by a mass loss of rapidly leaving matrix compounds, demonstrated by a narrow and rapid peak (Fig. 4). In this event, the DTG peak temperature indicated that the reaction occurred faster at 310.86 °C (Table 2). The third event was characterized by a slow, broad-derivative peak reaction corresponding to the physical or chemical interactions with the sample matrix (Fig. 3).

The sample of corn and urea mixture, and extruded urea presented three stages (Table 2 and Fig. 3). None of the samples presented constant stability, which indicates the water loss caused by the presence of corn, included in the same proportion for both samples (26.8%).

In the sample composed of corn and urea, the decomposition started at approximately 103.5 °C and the extruded urea at 136.2 °C, with the loss of mass recorded by the two samples in proportions of 54.9% and 50.5%, respectively (Table 2).

Similar behavior was observed in the second stage on the DTG curve (Fig. 4) of samples of corn and urea mixture, and extruded urea (Fig. 3), but the temperature of the beginning of mass variation (Table 2) was higher for extruded urea (231.7 °C) than for the corn and urea mixture sample (228.3 °C).

The third stage showed similarities in mass decomposition (Table 2) in the sample corn and urea mixture, and extruded urea (15.6 and 14.6%, respectively) and in the DTG (Fig. 4) the derivative peak was more extensive when compared to the first steps.

## Discussion

Using the images obtained from SEM it is possible to morphologically differentiate that the extrusion process alters the original structures of urea (Fig. 1a and b) and corn (Fig. 1c and d), attributed to the thickened shape and to modifications such as the disappearance of the starch granules (Fig. 1h) which was possibly caused by high pressure, temperature, and humidity. The extrusion gelatinized the starch and involved the non-crystalline structure of urea, which formed a complex structure. These characteristics of extruded urea make N release slower and more gradual (Fig. 2), which may improve utilization by rumen bacteria^2^ associated with the energy source of corn starch, can increase the synthesis of microbial proteins in the ruminant animals.

The rapid N solubilization of the maize and urea sample is evidenced by the maintenance of the original forms obtained by SEM images (Fig. 1e and f) and about 90% of the sample nitrogen is represented by fraction A, which is the non-protein soluble nitrogen from urea (Table 1). This behavior indicates that carbohydrate must be rapidly soluble and in sufficient amount to synchronize with NH_3_ and promote microbial protein synthesis. The constancy of N release at other times are contributions from fractions B1, B2, and B3 of corn N (Table 1), which are more slowly degraded fractions.

**Table 1:** Nutritional characterization of the mixture of ground corn and urea, and extruded urea.

Components (g/kg DM)	Mixture	Extruded urea
Dry matter (g/kg)	961.4	961.0
Organic matter	995.4	995.7
Crude protein (n-total × 6,25)	2040.4	2029.7
NPN (Fraction A)	292.3	298.1
CP from NNP (Fraction A)	1826.7	1863.3
N soluble (Fraction B1)	17.1	13.3
CP from N (Fraction B1)	106.9	83.2
N-True protein (Fraction B2)	8.83	5.22
CP from N (Fraction B2)	55.2	32.7
N-True protein (Fraction B3)	2.2	2.4
CP from N (Fraction B3)	13.7	15.0
N insoluble neutral detergent	7.9	7.8
N insoluble acid detergent (Fraction C)	6.1	5.7
CP from N (Fraction C)	37.9	35.6
Ethereal extract	32.5	31.6
Total carbohydrates	271.1	278.4
Non-fibrous carbohydrates	194.5	218.0
Neutral detergent fiber	52.0	32.5
Acid detergent fiber	21.6	18.3

The reduction in the rate of nitrogen solubilization in the aqueous medium of the extruded urea sample (Fig. 2) with even fraction A representing 90% of the nitrogen present in the sample (Table 1), can be evidenced by the grouping of structures observed in Fig. 1 (g and h), which configures a structural change of starch and urea after the extrusion process. Most of the N present in the samples belonging to the A fraction of the protein, also justifies the peak of N release that around 30 min. The constant N release pattern of this sample, observed up to 360 min, would allow rumen free ammonia to be used by rumen microorganisms along with rumen degradable carbohydrates at different speeds to synthesize microbial protein and prevent NH_3_ losses.

The rate of N release should be dependent on the rate of degradation of the carbohydrate source, where ammonia production in synergism with ruminal content energy metabolism is a decisive factor in microbial protein formation^6^.

The excess of N due to the high solubility of urea (Fig. 2) may, besides causing toxicity, constitute an energy waste as it requires energy to eliminate excess ammonia in the blood, negatively impacting the environment, with health and economic implications^12^.

Protein utilization efficiency in ruminants can be improved by maintaining adequate NNP amounts and feeding management with the use of extruded urea in cattle diets. Up to the level of supplementation of 80 g/100 kg body weight of extruded urea (starea with protein equivalent 200% CP) there are no negative influences on the rumen environment^13^.

Industry has improved to facilitate the use of NNP in ruminant diets, such as the urea extrusion process, to prevent ammonia poisoning and increase the availability of rumen nitrogen for microbial synthesis^14^. The urea sample showed thermal stability to its melting point at 133.0°C^15^.

When less mass loss occurs in the urea sample, in stages three and four (Table 2), it demonstrates that the complex polymerization and depolymerization reactions of high molecular weight compounds occurred and continued^16^.

**Table 2:** Characterization of steps, temperature of onset of mass loss, temperature of the highest reaction rate, mass loss and residue of urea, ground corn, ground corn and urea mixture, and extruded urea in air atmosphere with heating ratio of 10 °C per min^−1^.

Sample	Stage	T_a_ (°C)^1^	T_b_ (°C)^2^	Weight loss (%)	Residue (%)
Urea	1	133.0	190.4	0.2	0.47
	2	180.5	206.0	72.9	
	3	213.9	243.1	13.4	
	4	257.0	288.3	11.8	
	5	305.1	369.2	1.9	
Ground corn grain	1	37.2	212.6	12.3	1.3
	2	225.6	310.8	61.2	
	3	372.5	496.6	23.6	
Mix corn and urea*	1	103.5	188.7	54.9	0.32
	2	228.3	296.2	31.07	
	3	335.7	513.9	15.62	
Extruded urea*	1	136.2	194.5	50.57	0.34
	2	231.7	294.9	34.32	
	3	336.2	524.5	14.65	

^1^Temperature obtained by Weight Loss temp method.

^2^DTG peak temperature.

*200% protein equivalent (700 g/kg urea, 268 g/kg ground whole corn and 32 g/kg sulfur flower).

The gaseous products generated by urea pyrolysis are between temperatures from 132 to 190 °C. Experimental results show that at 132.5 to 160 °C, urea was only involved in consuming reactions. Biuret and cyanuric acid formation reactions, rather than the decomposition reaction of urea, occupy the major part of urea consumption. Above 160 °C, the dominant reaction of cyanide passes to decomposition formation, while the conversion temperature point of the biuret is 170 °C. NH_3_ production fluctuates at 132.5–170 °C, but continues to rise between 170 and 190°C^17^. In atmosphere with 5% or 10% O_2_, the highest NH_3_ production was reached at 200 C^15^.

The instability of the sample of ground whole corn was characterized by the loss of water, possibly by starch hydroxyls (Table 2). The second stage was characterized by a mass loss of rapidly leaving matrix compounds (Fig. 4), possibly by the decomposition of larger chain carbons and there were few physical or chemical interactions between the exiting compound and the sample matrix^16^.

The presence of water influenced the thermal stability of the corn and urea mixture sample, as it acts as a plasticizer in starch crystals, which may decrease the glass transition temperature and consequently the melting temperature of the crystals^18^ as evidenced in the whole corn sample (Fig. 3).

The delay in the onset of extruded urea decomposition and lower mass loss is possibly due to starch gelatinization, which after this process loses its initial structural organization, hydrogen bonds are broken, with crystalline fusion^3^, being the degree of gelatinization, higher for extruded foods, as the temperature used in the process is higher, up to 250 °C versus 60–95°C^19^. In addition, cornmeal produced by the dry milling process of maize grain has in its composition a higher proportion of starch, between 50 and 55%, 10% protein, 1% lipid^20^.

In the second stage on the DTG curve (Fig. 4) the temperature of the beginning of mass variation (Table 2) was higher for extruded urea which indicates that the extrusion process delays mass loss.

The characterization of extruded urea provides a detailed behavior of the product during its use, which allows its inclusion according to the characteristics of the animal and different nutritional management.

## Conclusions

We recommend the extrusion process to reducing N solubilization of nitrogen products and increasing nutritional quality of end product. The extrusion process of urea and corn to obtain extruded urea (starea) modifies the structure, increase the quality by starch gelatinization and complexation with the crystals of urea, and preventing rapid solubilization of nitrogen.

## Materials and methods

### Samples and nutritional characterization

Samples of two products denominated corn and urea mixture were used, corresponding to the mixture of ingredients before extrusion and extruded urea—Starea (Amiréia Pajoara 200S). The production process of extruded urea consists of the complexation of urea with a starch molecule gelatinized, using pressure, temperature and humidity, thus obtaining the starea. The two samples, with a protein equivalent of 200%, were composed of livestock urea, ground corn grain, and sulfur flower in the proportions of 70%, 26.8%, and 3.2%, respectively^3^.

The two ingredients were sent for chemical analysis in the original particle size in order to test the processing effect. Dry matter (DM; Table 1) was determined by drying in an oven at 105 °C overnight (method 930.15^21^). The total nitrogen content was quantified by the Kjeldahl method using the Tecnal TE-036/1 distiller (Tecnal, Piracicaba, Brazil) (method 976.05^21^). The determination of ether extract was performed on an Ankom XT 10 extractor apparatus (Ankon Technology, NY, USA). The ash content was determined by muffle incineration (method 942.05^21^) and the organic matter (OM) content was calculated by the difference between 100 and the ash percentage. To determine the neutral detergent fiber (NDF)^22^ content, thermostable α-amylase (Termamyl 120 L Sigma-Aldrich, 3050 Spruce Street, Saint Louis, MO, USA) and the results were expressed with residual ash. Were determined the percentage of total carbohydrates (TC) ^23^ and non-fibrous carbohydrates (NFC)^24^.

Protein fractions were determined according to the methodology^25^ described with fraction A corresponding to NPN, B1 corresponding to true soluble protein, B2 o N to rumen-insoluble true protein, but not bound to NDF, B3 corresponding to potentially available N-linked fiber (insoluble nitrogen in neutral detergent), and C the unavailable portion, linked to lignin (acid detergent insoluble nitrogen).

### Scanning electron microscopy (SEM) observations

The study of sample morphology (Fig. 1) was performed by scanning electron microscopy (SEM) measurements on JEOL model JSM 6380LV equipment at Physics Institute of Federal University of Mato Grosso do Sul (INFI/UFMS).

Samples in the original form of urea, ground corn grain, corn and urea mixture, and extruded urea were dispersed on double-sided carbon tape for attachment to the top of a copper stub. The set was taken into the sputtering chamber (Denton Vacuum, Desk III model) for the deposition of a thin gold layer on the surface of the dust particles to obtain a better SEM image resolution and increased conductivity of the sample to avoid the effects of electronic loading that impair image resolution. For the analysis, a voltage of 15 kV, working distance (WD) 12 mm, and spot size 20 were used. Readings were taken and images were recorded at 27 and 2000 times increases for comparative morphology purposes.

### In vitro nitrogen solubility

To estimate nitrogen (N) release in different products, the Kjeldahl method was used to determine total nitrogen, with modifications^17^.

Initially 2 g of the sample composed of ground corn and urea and extruded urea (starea) were packed in glassware of 500 mL capacity. Then 200 mL of distilled water was added and the samples were taken to the water bath (39.5 °C) in stirring. The total analysis time was 360 min, and a 2 mL aliquot (in triplicate) was removed from the supernatant at times 0, 30, 60, 120, 180, 240, 300 and 360 min, according to methodology described to Ítavo et al.^6^.

These aliquots were packed into digestion tubes containing 5 mL of 2 N potassium hydroxide (KOH) and 13 mL of distilled water. Subsequently, the digestion tubes were taken to the nitrogen distiller (TECNAL—TE-036/1). In the same equipment an Erlenmeyer containing 10 mL of 2% boric acid was also coupled and then distillation occurred until obtaining 75 mL of solution.

Finally, titrations of the recovered solutions were made in the distiller, with 0.005 N of hydrochloric acid (HCl) used until the turning point of the solutions was obtained. As with distillation, titration was also performed in triplicate for each product and for each analysis time. A blank (distilled water only) was also performed at the beginning of each analysis time to verify equipment contamination.

The data obtained with the titrations were submitted to the following equation: N concentration (g/dL) = HCl volume used x HCl correction factor × 0.005 × 0.014 × 100/2.

### Thermogravimetry and derived thermogravimetry measurements

The thermogravimetry (TG) and derivative thermogravimetry (DTG) curves of the original urea, ground whole corn, corn and urea mixture, and extruded urea samples were determined with a TGA Q50 V20.13 thermogravimetric analyzer, Build 39 (New Castle, USA), at Chemical Institute of Federal University of Mato Grosso do Sul. About 6.0 mg of each sample was weighed on platinum cell and the tests were carried out in a synthetic air atmosphere, with a heating rate of 10 °C min^−1^, from room temperature to 900 °C. The temperatures at which the decomposition rate of the sample was maximum (Td) were obtained through the DTG curves. Data were analyzed using Advantage/Universal Analyzes Software v5.5.24 available from TA Instruments.

### Ethics statement

All plant studies and sampling were carried out in accordance with relevant institutional, national or international guidelines. We used ground corn grain (*Zea mays*) commercial.
